# Non-cardiac chest pain patients in the emergency department: Do physicians have a plan how to diagnose and treat them? A retrospective study

**DOI:** 10.1371/journal.pone.0211615

**Published:** 2019-02-01

**Authors:** Maria M. Wertli, Tenzin D. Dangma, Sarah E. Müller, Laura M. Gort, Benjamin S. Klauser, Lina Melzer, Ulrike Held, Johann Steurer, Susann Hasler, Jakob M. Burgstaller

**Affiliations:** 1 Division of General Internal Medicine, Bern University Hospital, University of Bern, Bern, Switzerland; 2 Horten Centre for Patient Oriented Research and Knowledge Transfer, University of Zurich, Zurich, Switzerland; 3 Kantonsspital Winterthur, Department of General Internal Medicine Outpatient Clinic, Winterthur, Switzerland; University Magna Graecia of Catanzaro, ITALY

## Abstract

**Background:**

Non-cardiac chest pain is common and there is no formal recommendation on what diagnostic tests to use to identify underlying diseases after an acute coronary syndrome has been ruled out.

**Objective:**

To evaluate the diagnostic tests, treatment recommendations and initiated treatments in patients presenting with non-cardiac chest pain to the emergency department (ED).

**Methods:**

Single-center, retrospective medical chart review of patients presenting to the ED. Included were all medical records of patients aged 18 years and older presenting to the ED with chest pain and a non-cardiac discharge diagnosis between January 1, 2009 and December 31, 2011. Information on the diagnosis, diagnostic tests performed, treatment initiated and recommendation for further diagnostic testing or treatment were extracted. The primary outcomes of interest were the final diagnosis, diagnostic tests, and treatment recommendations. A formal ACS rule out testing was defined as serial three troponin testing.

**Results:**

In total, 1341 ED admissions for non-cardiac chest pain (4.2% of all ED admissions) were analyzed. Non-specific chest pain remained the discharge diagnosis in 44.7% (n = 599). Identified underlying diseases included musculoskeletal chest pain (n = 602, 44.9%), pulmonary (n = 30, 2.2%), GI-tract (n = 35, 2.6%), or psychiatric diseases (n = 75, 5.6%). In 81.4% at least one troponin test and in 89% one ECG were performed. A formal ACS rule out troponin testing was performed in 9.2% (GI-tract disease 14.3%, non-specific chest pain 14.0%, pulmonary disease 10.0%, musculoskeletal chest pain 4.7%, and psychiatric disease 4.0%). Most frequently analgesics were prescribed (51%). A diagnostic test with proton pump inhibitor (PPI) was prescribed in 20% (mainly in gastrointestinal diseases). At discharge, over 72 different recommendations were given, ranging from no further measures to extensive cardiac evaluation.

**Conclusion:**

In this retrospective study, a formal work-up to rule out ACS was found in a minority of patients presenting to the ED with chest pain of non-cardiac origin. A wide variation in diagnostic processes and treatment recommendations reflect the uncertainty of clinicians on how to approach patients after a cardiac cause was considered unlikely. Panic and anxiety disorders were rarely considered and a useful PPI treatment trial to diagnose gastroesophageal reflux disease was infrequently recommended.

## Introduction

The top priority in patients presenting with chest pain to the emergency department (ED) is to rule out a potentially life-threatening disease such as an acute coronary syndrome (ACS), pulmonary embolism, aortic dissection, or pneumonia. After a thorough diagnostic work-up, an acute myocardial ischemia can be ruled out for 60% to 90% of patients presenting with chest pain [1–4]. While in specialized units, including cardiac care units and intensive care units, the proportion of patients with ACS may be higher [5], the percentage of patients in the ED with ACS decreased in the US from 23.6% in 1999–2000 to 13.0% in 2007–2008 [6]. When no specific disease causing the chest pain can be identified, patients are usually discharged with the diagnosis of non-cardiac chest pain (NCCP).

Patients with NCCP can be categorized in patients with and without an identifiable underlying disease (i.e. non-specific chest pain). It has been suggested that up to 50% of the patients discharged with NCCP have an underlying gastrointestinal reflux [7] or a psychiatric disease [8, 9]. Further, chest pain is frequently the result of musculoskeletal diseases [10]. Whereas the mortality rates among patients discharged with NCCP from the ED is low [11], 90% complained of persisting symptoms and impaired quality of life at a 4-year follow-up [12]. Despite normal coronary angiograms, 44% of patients with NCCP still believed they suffer from an underlying cardiac disease and 50% reported limitations in performing their daily activities [13]. Therefore, a primary focus on ruling out cardiovascular disease in patients with NCCP may result in overtesting without improving the patients’ confidence. Further, elevated troponin test results can be found in patients without chest pain or ischemic electrocardiographic changes and, in a retrospective study, elevated troponin test results had no clinical utility but resulted in downstream testing [14].

Therefore, the clinical challenge is to determine which diagnostic tests to apply in patients with chest pain after a cardiac disease has been ruled out in order to discriminate between patients with non-specific chest pain and other underlying diseases presenting with NCCP. For example, a high dose proton pump inhibitor (PPI) treatment trial may be useful to identify patients with underlying gastroesophageal reflux disease (GERD) and screening tools may identify patients with an underlying panic or anxiety disorder [15]. To date, the diagnostic processes and the treatment recommendations in patients discharged from the ED with a diagnosis of NCCP are poorly investigated and mainly based on the physicians’ personal beliefs and experiences.

The objective of this retrospective study was to gather knowledge about the diagnostic steps in the ED and the treatment recommendations for patients discharged from the ED with a diagnosis of NCCP. We analyzed the frequency of discharge diagnoses, the performed diagnostic tests, the initiated treatments, and the treatment recommendations. We hypothesized that the majority of patients were discharged with a musculoskeletal or non-specific disease and the diagnostic assessment focused mainly on ruling out of an ACS. Further, we hypothesized that a high-dose PPI treatment trial to identify patients with GERD related chest pain was infrequently used and psychiatric diseases rarely considered.

## Methods

Single-center, retrospective medical chart review of patients presenting to one of the ten largest hospitals in Switzerland, the Cantonal Hospital Winterthur, between January 1, 2009 and December 31, 2011. The study period was chosen because an outpatient clinic opened in 2012 and therefore, many patients eligible for this study were potentially treated elsewhere. The hospital is affiliated to the University of Zurich and covers the medical services for approximately 200'000 persons (15 percent of the inhabitants of the canton Zürich).

### Patient selection

Potentially eligible medical records were identified by using prespecified diagnostic German International Classification of Disease Version 10 (ICD-10-GM) codes coded by ED physicians: R06.4 (hyperventilation), R07.1 (chest pain when breathing), R07.2 (precordial pain), R07.3 (other chest pain), and R07.4 (chest pain not further specified).

### Eligibility criteria

Included were all medical records of patients age 18 years and older presenting to the ED with chest pain and a non-cardiac discharge diagnosis between January 1, 2009 and December 31, 2011. Excluded were patients with chest pain of cardiovascular origin, pregnant women, trauma patients or life-threatening conditions, malignant disease, current fracture, renal replacement therapy or severe kidney failure (creatinine clearance of less than 30ml/min/1.73m^2^) as well as patients with incapacitation or records of patients which opted out of releasing their records for scientific purposes.

### Data extraction procedure

All records identified by this search were screened by two researchers (TD, SM) for inclusion or exclusion. In case of uncertainty, the records were discussed with the principal investigator (MW) and disagreement was resolved within the research group. Each patient included in the study was assigned a unique de-identified number. We defined the first presentation for chest pain to the ED as the index consultation for the first episode. During the following three months each presentation (to the ED, outpatient consultation, hospitalization) was considered potentially related to the index consultation and was defined as a follow-up consultation. Presentations to the ED or hospitalizations after more than three months due to chest pain were defined as a new index visit of a second episode.

Variables of interest were predefined and the extraction form was pilot-tested in 20 records. To ensure high quality in the data extraction, TD and SM were trained and monitored by MW and an extraction manual was used. We extracted information on general characteristics (age, gender), cardiovascular risk factors, signs and symptoms at presentation, preexisting comorbidities, medications, clinical findings at presentation, blood analyses, ECG, imaging studies, coronary angiography, non-invasive testing (e.g. treadmill testing, cardiac scintigraphy, echocardiography) and other tests/investigations. Further information on discharge medications, discharge diagnosis, recommended procedures / investigations after discharge were extracted.

### Study endpoint

The main study endpoints of interests were the final diagnosis, performed diagnostic tests, and treatment recommendations. The final diagnosis was based on the discharge diagnosis extracted from the discharge letters. In patients with re-visits to an outpatient clinic or the ED any additional follow-up assessment and reports were reviewed and screened for changes in the discharge diagnosis. In patients with differences between the discharge diagnosis and the diagnosis on follow-up visits, the final diagnosis was adjudicated by a research committee (JS, UH, JB, MW)–blinded to the details of index visit–based on the results of the follow-up evaluation and records of re-hospitalizations. Each final diagnosis was assigned to one of the five categories: musculoskeletal chest pain, gastrointestinal chest pain, pulmonary chest pain, chest pain in psychiatric diseases, and non-specific chest pain.

We defined a formal ACS rule out testing as serial troponin tests performed at presentation, a second (after 3 to 6 hours), and a third (beyond 6 hours) [16]. Additional endpoints were: recommendations on further evaluation after discharge, and re-visits to the ED.

### Data quality and statistical analysis

The quality of the data extraction was assessed by a researcher not involved in the extraction process (BK). In total, six predefined parameters (troponin test result, pain reproducible by movement, coronary angiography, recommendation for further diagnostic evaluation, recommendation for further treatment, and the discharge diagnosis) in 379 ED visits were reviewed. The quality of data extraction was high with an error rate of 5.4% (95% CI 4.5–6.4).

We calculated median and interquartile ranges for continuous variables, numbers and percentages of total for binary or categorical variables. A chi-squared test was used for group comparisons for categorical variables and Kruskal-Wallis test was used for continuous variables between groups. Differences between the diagnostic categories were visualized using bar plots. All analyses were performed with the statistical software R [17].

### Ethical review board approval

Due to the retrospective nature of the study, data extraction did not interfere or influence the treatment of patients. The study was approved by the independent Ethics Committee of the Canton Zurich, Switzerland (KEK-ZH number 2014–0506, approved in December 2014) and complied with international standards including the declaration of Helsinki, good clinical practice, and the Swiss law for research in human subjects.

## Results

Out of 31,902 visits to the ED, 2,438 records with the ICD-10 codes R07.1–4 were screened and 1,341 ED admissions for non-cardiac chest pain (4.2%, **Table 1**) were finally analyzed. The main discharge diagnoses were musculoskeletal chest pain (n = 602, 45%) and non-specific chest pain (n = 599, 45%). Musculoskeletal diagnosis were mainly non-specific related to the chest wall (90%) or to the spine (3%). Specific musculoskeletal diagnosis were found in a few patients (fractured rip n = 5, late onset rheumatoid arthritis n = 1, and contusion n = 3). In a small proportion of patients the diagnostic work-up resulted in a pulmonary (n = 30, 2%), GI-tract (n = 35, 2%), or psychiatric diseases (n = 75, 6%).

**Table 1:** Baseline characteristics.

	Overall	MSD	Non-specific	Pulmonary	GI Tract	Psychiatric	p-value
Number of patients	1341	602	599	30	35	75	
% of the population	100.0	44.9	44.7	2.2	2.6	5.6	
Age	46.0 [33.0, 60.0]	40.5 [30.0, 55.0]	49.0 [38.0, 64.0]	60.0 [33.0, 68.0]	56.0 [41.5, 63.0]	43.0 [29.5, 53.0]	**<0.001**
Male	604 (45.0)	265 (44.0)	269 (44.9)	12 (40.0)	13 (37.1)	45 (60.0)	0.08
Profession							**<0.001**
Employee/white collar	295 (22.0)	162 (26.9)	107 (17.9)	5 (16.7)	2 (5.7)	19 (25.3)	
Blue collar	137 (10.2)	74 (12.3)	51 (8.5)	1 (3.3)	4 (11.4)	7 (9.3)	
Disabled	21 (1.6)	13 (2.2)	5 (0.8)	1 (3.3)	1 (2.9)	1 (1.3)	
Non-working	133 (9.9)	71 (11.8)	41 (6.8)	4 (13.3)	4 (11.4)	13 (17.3)	
Retired	179 (13.3)	64 (10.6)	94 (15.7)	7 (23.3)	5 (14.3)	9 (12.0)	
Unknown	576 (43.0)	218 (36.2)	301 (50.3)	12 (40.0)	19 (54.3)	26 (34.7)	
Marital status							**<0.001**
Divorced	146 (10.9)	69 (11.5)	67 (11.2)	4 (13.3)	3 (8.6)	3 (4.0)	
No relationship	289 (21.6)	157 (26.1)	99 (16.5)	8 (26.7)	3 (8.6)	22 (29.3)	
Relationship	8 (0.6)	2 (0.3)	4 (0.7)	0 (0.0)	1 (2.9)	1 (1.3)	
Married	781 (58.2)	334 (55.5)	368 (61.4)	13 (43.3)	22 (62.9)	44 (58.7)	
Widowed	81 (6.0)	25 (4.2)	41 (6.8)	5 (16.7)	6 (17.1)	4 (5.3)	
Unknown	36 (2.7)	15 (2.5)	20 (3.3)	0 (0.0)	0 (0.0)	1 (1.3)	
Presentation at ER							**<0.001**
By ambulance	200 (14.9)	70 (11.6)	99 (16.5)	3 (10.0)	6 (17.1)	22 (29.3)	
No	690 (51.5)	370 (61.5)	247 (41.2)	18 (60.0)	17 (48.6)	38 (50.7)	
Unknown	451 (33.6)	162 (26.9)	253 (42.2)	9 (30.0)	12 (34.3)	15 (20.0)	
Referral							**<0.001**
Self-referral	1105 (82.4)	524 (87.0)	466 (77.8)	23 (76.7)	26 (74.3)	66 (88.0)	
Physician referral	232 (17.3)	78 (13.0)	130 (21.7)	6 (20.0)	9 (25.7)	9 (12.0)	
Not reported	4 (0.3)	0 (0.0)	3 (0.5)	1 (3.3)	0 (0.0)	0 (0.0)	
**CVD risk factors**							
BMI	25.8 [23.2, 29.1]	25.6 [23.0, 28.8]	26.1 [23.9, 29.4]	24.8 [23.3, 26.6]	28.4 [24.8, 32.2]	25.4 [22.0, 28.5]	**0.03**
Smoking: current	254 (18.9)	109 (18.1)	116 (19.4)	3 (10.0)	9 (25.7)	17 (22.7)	**<0.001**
Stopped	153 (11.4)	45 (7.5)	94 (15.7)	5 (16.7)	5 (14.3)	4 (5.3)	
Never	255 (19.0)	96 (15.9)	128 (21.4)	5 (16.7)	13 (37.1)	13 (17.3)	
Not reported	679 (50.6)	352 (58.5)	261 (43.6)	17 (56.7)	8 (22.9)	41 (54.7)	
Family history of CVD	210 (15.7)	80 (13.3)	109 (18.2)	3 (10.0)	9 (25.7)	9 (12.0)	**<0.001**
No	332 (24.8)	118 (19.6)	174 (29.0)	10 (33.3)	15 (42.9)	15 (20.0)	
Not reported	799 (59.6)	404 (67.1)	316 (52.8)	17 (56.7)	11 (31.4)	51 (68.0)	
Known CVD	459 (34.2)	151 (25.1)	258 (43.1)	11 (36.7)	16 (45.7)	23 (30.7)	**<0.001**
No	630 (47.0)	317 (52.7)	251 (41.9)	15 (50.0)	15 (42.9)	32 (42.7)	
Not reported	252 (18.8)	134 (22.3)	90 (15.0)	4 (13.3)	4 (11.4)	20 (26.7)	
Previous acute MI	116 (8.7)	31 (5.1)	73 (12.2)	1 (3.3)	7 (20.0)	4 (5.3)	**<0.001**
No	1023 (76.3)	482 (80.1)	438 (73.1)	25 (83.3)	23 (65.7)	55 (73.3)	
Not reported	89 (14.8)	88 (14.7)	4 (13.3)	5 (14.3)	16 (21.3)	89 (14.8)	
PAD	11 (0.8)	3 (0.5)	7 (1.2)	0 (0.0)	0 (0.0)	1 (1.3)	0.66
No	1101 (82.1)	505 (83.9)	484 (80.8)	26 (86.7)	29 (82.9)	57 (76.0)	
Not reported	229 (17.1)	94 (15.6)	108 (18.0)	4 (13.3)	6 (17.1)	17 (22.7)	
History of stroke	26 (1.9)	6 (1.0)	13 (2.2)	3 (10.0)	2 (5.7)	2 (2.7)	0.015
No	1109 (82.7)	505 (83.9)	494 (82.5)	24 (80.0)	29 (82.9)	57 (76.0)	
Not reported	206 (15.4)	91 (15.1)	92 (15.4)	3 (10.0)	4 (11.4)	16 (21.3)	
Diabetes mellitus	69 (5.1)	22 (3.7)	38 (6.3)	1 (3.3)	1 (2.9)	7 (9.3)	**0.032**
No	1107 (82.2)	520 (86.4)	472 (78.8)	26 (86.7)	30 (85.7)	56 (74.7)	
Not reported	168 (12.5)	60 (10.0)	89 (14.9)	3 (10.0)	4 (11.4)	12 (16.0)	
**Medication use**							
Diabetes mellitus therapy							0.37
Diet	4 (0.3)	1 (0.2)	3 (0.5)	0 (0.0)	0 (0.0)	0 (0.0)	
Oral antidiabetic drugs	47 (3.5)	17 (2.8)	25 (4.2)	1 (3.3)	0 (0.0)	4 (5.3)	
Insulin	18 (1.3)	4 (0.7)	10 (1.7)	0 (0.0)	1 (2.9)	3 (4.0)	
Acetylsalicylic acid use	218 (16.3)	64 (10.6)	128 (21.4)	5 (16.7)	9 (25.7)	12 (16.0)	**<0.001**
No	995 (74.2)	477 (79.2)	416 (69.4)	23 (76.7)	24 (68.6)	55 (73.3)	
Not reported	128 (9.5)	61 (10.1)	55 (9.2)	2 (6.7)	2 (5.7)	8 (10.7)	
Statin use	178 (13.3)	51 (8.5)	107 (17.9)	3 (10.0)	11 (31.4)	6 (8.0)	**<0.001**
No	1031 (76.9)	490 (81.4)	434 (72.5)	25 (83.3)	22 (62.9)	60 (80.0)	
Not reported	132 (9.8)	61 (10.1)	55 (9.2)	2 (6.7)	2 (5.7)	8 (10.7)	
Antihypertensive therapy	357 (26.6)	112 (18.6)	205 (34.2)	10 (33.3)	11 (31.4)	19 (25.3)	**<0.001**
No	854 (63.7)	429 (71.3)	337 (56.3)	18 (60.0)	22 (62.9)	48 (64.0)	
Not reported	130 (9.7)	61 (10.1)	57 (9.5)	2 (6.7)	2 (5.7)	8 (10.7)	
PPI	162 (12.1)	49 (8.1)	89 (14.9)	3 (10.0)	12 (34.3)	9 (12.0)	**<0.001**
No	1047 (78.1)	491 (81.6)	452 (75.5)	25 (83.3)	21 (60.0)	58 (77.3)	
Not reported	132 (9.8)	62 (10.3)	58 (9.7)	2 (6.7)	2 (5.7)	8 (10.7)	
Analgesics	194 (14.5)	94 (15.6)	80 (13.4)	6 (20.0)	5 (14.3)	9 (12.0)	0.91
No	1020 (76.1)	449 (74.6)	463 (77.3)	22 (73.3)	28 (80.0)	58 (77.3)	
Not reported	127 (9.5)	59 (9.8)	56 (9.3)	2 (6.7)	2 (5.7)	8 (10.7)	
Antipsychotics	175 (13.0)	59 (9.8)	93 (15.5)	4 (13.3)	5 (14.3)	14 (18.7)	0.17
No	1035 (77.2)	481 (79.9)	450 (75.1)	24 (80.0)	27 (77.1)	53 (70.7)	
Not reported	131 (9.8)	62 (10.3)	56 (9.3)	2 (6.7)	3 (8.6)	8 (10.7)	

Values in median [IQR], n (%); p-values refer to all columns except for the overall. A chi-squared test was used for all variables except Age and BMI where Kruskal-Wallis was used.

MSD, musculoskeletal diseases; GI, gastrointestinal; CVD, cardiovascular disease; PAD, peripheral arterial disease; BMI, body mass index; PPI, proton pump inhibitor; Gyn, gynecological; not reported, no information available in the electronical records.

### Baseline presentation

The majority of patients were female (55%), married (58%), and the median age was 46 years (IQR 33–60, **Table 1**). Cardiovascular disease was known in 34.2% (previous acute myocardial infarction in 8.7%), a history of peripheral arterial disease in 0.8%, stroke in 1.9%, and diabetes mellitus in 5.1%. Overall, 32% of the patients met the definition for multimorbidity (≥ two known diseases). Further details are summarized in **Table 1** and **S1 Table.**

### ED evaluation

**Fig 1:**
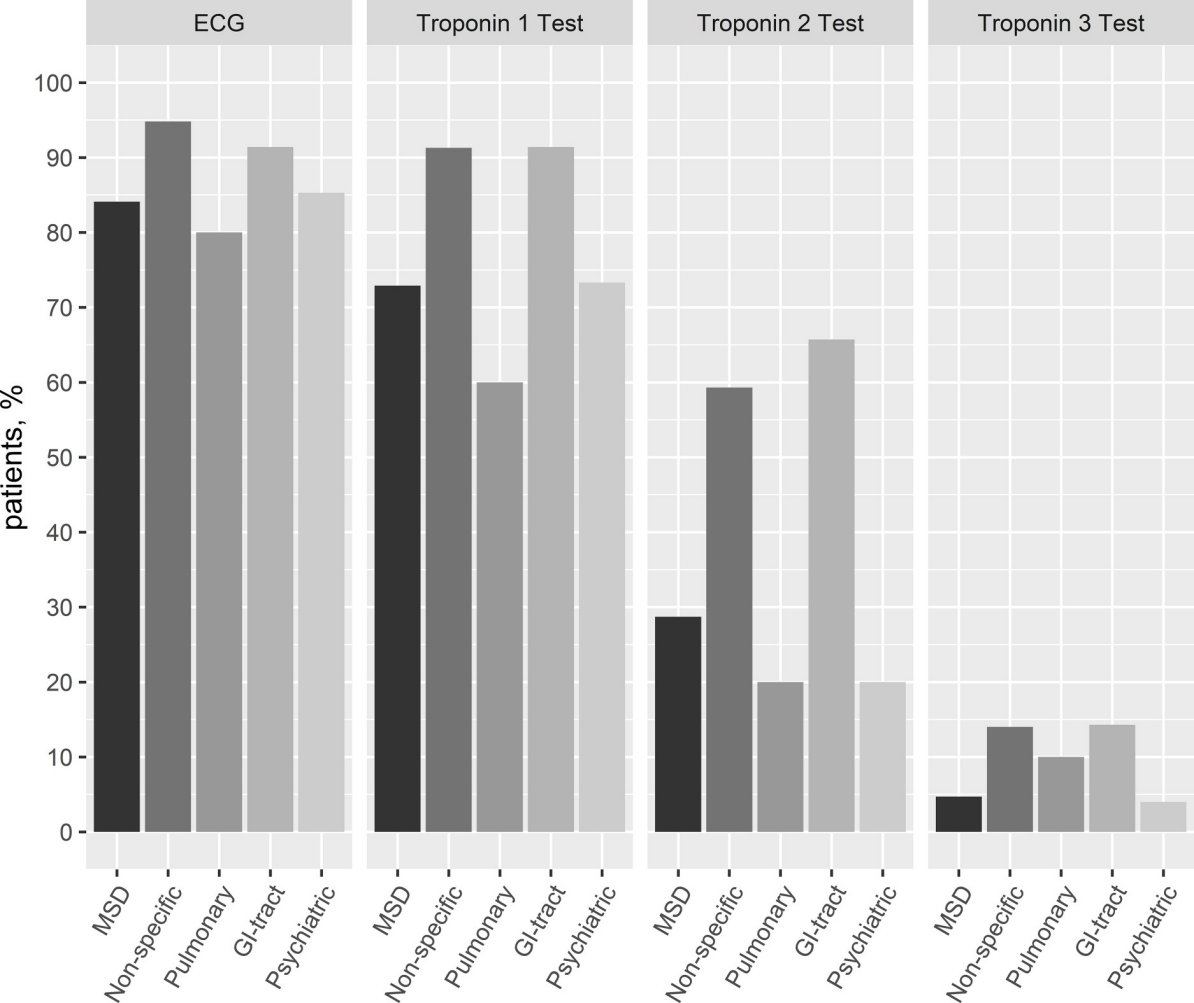
Initial ECG and troponin testing in patients non-cardiac chest pain groups. MSD, musculoskeletal chest pain; non-specific, non-specific chest pain; pulmonary, pulmonary diseases; GI-tract, gastrointestinal tract related chest pain; Psychiatric, chest pain related to psychiatric conditions.

Diagnostic evaluation was mainly performed on an outpatient basis (90.2%, **Table 2**). The diagnostic work-up with regards to vital signs was comparable in all categories: blood pressure measurements in 91.6%, oxygen saturation or the respiratory rate in 74.8%, and body temperature in 51.1%. Overall, in 89% of the patients an ECG, in 91.9% at least one blood analysis, and in 81.4% at least one troponin test was performed. **Fig 1** shows the proportion of patients for each diagnostic group with initial and follow-up troponin testing. The proportion of initial troponin testing varied between 90% (non-specific chest pain 90.3% and GI-diseases 90.4%) and below 75% (musculoskeletal chest pain 72.9%, pulmonary diseases 60%, and psychiatric diseases 73.3%). A second follow-up troponin test was performed in 42.7% and a formal ACS rule out with a third troponin test in 9.2% (**Table 2** and **S2 Table**). The formal ACS rule out testing was performed in patients with GI-tract diseases (14.3%), non-specific chest pain (14.0%), pulmonary diseases (10.0%), musculoskeletal chest pain (4.7%), and psychiatric diseases (4.0%).

**Table 2:** Diagnostic evaluations in patients with non-cardiac chest pain.

	Overall	MSD	Non-specific	Pulmonary	GI-tract	Psychiatric	p
Patients: n	1341	602	599	30	35	75	
Outpatient evaluation	1209 (90.2)	577 (95.8)	517 (86.3)	19 (63.3)	28 (80)	68 (90.7)	<0.001
Inpatient evaluation	132 (9.8)	25 (4.2)	82 (13.7)	11 (36.7)	7 (20.0)	7 (9.3)	
Intensive care unit	6 (0.4)	1 (0.2)	3 (0.5)	2 (6.7)	0 (0.0)	0 (0.0)	<0.001
**Vital signs**							
Arterial BP recorded	1229 (91.6)	556 (92.4)	544 (90.8)	26 (86.7)	32 (91.4)	71 (94.7)	0.59
BP measurement both sides	904 (67.4)	395 (65.6)	416 (69.4)	23 (76.7)	21 (60.0)	49 (65.3)	0.38
SO2 or respiratory rate	1003 (74.8)	464 (77.1)	431 (72.0)	24 (80.0)	24 (68.6)	60 (80.0)	0.17
Temperature	685 (51.1)	311 (51.7)	302 (50.4)	17 (56.7)	16 (45.7)	39 (52.0)	0.91
**Lab and ECG testing**							
Laboratory (any)	1233 (91.9)	527 (87.5)	581 (97.0)	26 (86.7)	34 (97.1)	65 (86.7)	**<0.001**
ECG	1194 (89.0)	506 (84.1)	568 (94.8)	24 (80.0)	32 (91.4)	64 (85.3)	**<0.001**
Troponin test at presentation	1091 (81.4)	439 (72.9)	547 (91.3)	18 (60.0)	32 (91.4)	55 (73.3)	**<0.001**
Not measured	250 (18.6)	163 (27.1)	52 (8.7)	12 (40.0)	3 (8.6)	20 (26.7)	
Troponin 2° Test‡	572 (42.7)	173 (28.7)	355 (59.3)	6 (20.0)	23 (65.7)	15 (20.0)	**<0.001**
Troponin 3° Test‡	123 (9.2)	28 (4.7)	84 (14.0)	3 (10.0)	5 (14.3)	3 (4.0)	**0.001**
**Additional tests: n (%)**							
Tread mill test	35 (2.6)	8 (1.3)	26 (4.3)	0 (0.0)	1 (2.9)	0 (0.0)	**0.008**
Echocardiography	65 (4.8)	18 (3.0)	38 (6.3)	1 (3.3)	2 (5.7)	6 (8.0)	0.06
MIBI scintigraphy	9 (0.7)	0 (0.0)	9 (1.5)	0 (0.0)	0 (0.0)	0 (0.0)	**0.02**
Coronary angiography	40 (3.0)	8 (1.3)	30 (5.0)	0 (0.0)	1 (2.9)	1 (1.3)	**0.003**
Chest x-ray	789 (58.8)	339 (56.3)	383 (63.9)	22 (73.3)	21 (60.0)	24 (32.0)	**<0.001**
Chest CT scan	114 (8.5)	46 (7.6)	51 (8.5)	8 (26.7)	5 (14.3)	4 (5.3)	**0.003**
Abdominal CT scan	13 (1.0)	4 (0.7)	8 (1.3)	0 (0.0)	1 (2.9)	0 (0.0)	0.44
Abdominal sonography	45 (3.4)	10 (1.7)	26 (4.3)	5 (16.7)	4 (11.4)	0 (0.0)	**<0.001**
Gastroscopy	13 (1.0)	1 (0.2)	7 (1.2)	0 (0.0)	4 (11.4)	1 (1.3)	**<0.001**
Pulmonary function test	11 (0.8)	4 (0.7)	6 (1.0)	0 (0.0)	0 (0.0)	1 (1.3)	0.88
Pleura sonography	5 (0.4)	2 (0.3)	1 (0.2)	2 (6.7)	0 (0.0)	0 (0.0)	**<0.001**
Patients requiring surgery	3 (0.2)	1 (0.2)	2 (0.3)	0 (0.0)	0 (0.0)	0 (0.0)	0.95
Other interventions	82 (6.1)	25 (4.2)	36 (6.0)	3 (10.0)	1 (2.9)	17 (22.7)	**<0.001**

‡ details on the time between baseline and follow-up testing are provided in **S2 Table**.

SO2, oxygen saturation

MSD, musculoskeletal diseases; GI, gastrointestinal; CVD, cardiovascular disease; PAD, peripheral arterial disease; CT, computer tomography; MIBI, methoxyisobutylisonitrile

Chi-squared tests were used for all variables.

**Fig 2:**
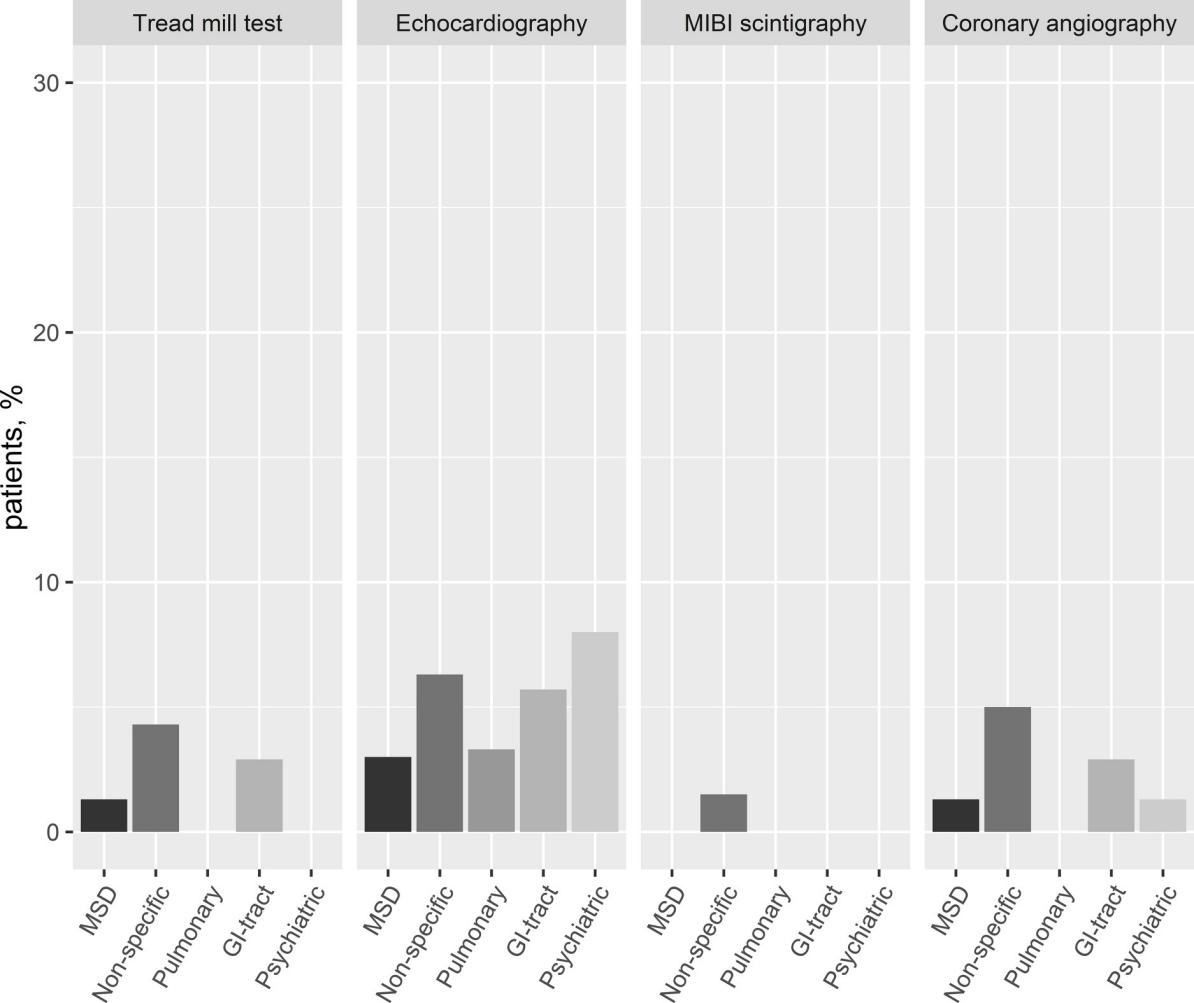
Additional non-invasive and invasive tests for cardiac diseases. MSD, musculoskeletal chest pain; non-specific, non-specific chest pain; pulmonary, pulmonary diseases; GI-tract, gastrointestinal tract related chest pain; Psychiatric, chest pain related to psychiatric conditions.

The most frequently performed additional diagnostic test was a chest x-ray (58.9%). Around 10% of all patients were hospitalized (0.4% in an intensive care unit). Additional evaluation using a tread mill test was performed in 2.6%, echocardiography in 4.8%, MIBI scintigraphy in 0.7%, and coronary angiography in 3.0%. **Fig 2** shows the differences in the use of these test for each diagnostic groups. In non-specific chest pain, tread mill tests, MIBI scintigraphy, and coronary angiography were most often performed. Echocardiography was most often performed in patients with a psychiatric discharge diagnosis.

### Recommendations at discharge and initiated treatments

Overall, 76 different recommendations at discharge were identified. In **Table 3** the most frequent recommendations and initiated treatments are summarized. In 17% of the patients further cardiac evaluation (treadmill testing 9.1%, least often coronary angiography 0.2%) was recommended followed by a GP follow-up or a GP initiated assessment/action (16.6%). In patients with non-specific chest pain cardiac evaluation and GP follow-up was most often recommended (26.5% and 20.8%, respectively). Whereas a psychiatric evaluation was recommended in very few patients (<1%), anxiolytics or psychological treatment was most often initiated in patients with psychiatric discharge diagnosis (18.7%).

**Table 3:** Summary of recommendations, initiated treatment and follow-up evaluations.

	overall	MSD	Non-specific	Pulmonary	GI-tract	Psychiatric	p
Number: n	1341	602	599	30	35	75	
**Recommendations at discharge (main): n (%)**							
Further imaging studies	21 (1.6)	8 (1.3)	9 (1.5)	3 (10)	1 (2.9)	0 (0)	<0.001
Further cardiac assessment	228 (17)	55 (9.1)	159 (26.5)	1 (3.3)	4 (11.4)	9 (12)	<0.001
Further gastroenterological assessment	42 (3.1)	5 (0.8)	27 (4.5)	0 (0)	6 (17)	4 (5.3)	<0.001
Psychiatric evaluation	12 (0.9	5 (0.8)	4 (0.7)	3 (10)	0 (0)	0 (0)	<0.001
Pneumological evaluation	19 (1.4)	7 (1.2)	8 (1.3)	1 (3.3)	0 (0)	3 (4)	
Other evaluations	25 (1.9)	10 (1.7)	7 (1.2)	1 (3.3)	4 (11.4)	3 (4)	<0.001
PPI treatment	30 (2.2)	6 (1.0)	16 (2.7)	0 (0)	7 (0.2)	1 (1.3)	
Anxiolytics / psychological treatment	45 (3.4)	11 (1.8)	19 (3.2)	1 (3.3)	0 (0)	14 (18.7)	
GP assessment or action	72 (5.4)	32 (6.3)	29 (4.8)	2 (6.7)	4 (11.4)	5 (6.7)	
GP follow-up	150 (11.2)	41 (6.8)	96 (16.0)	2 (6.7)	4 (11.4)	7 (9.3)	
Musculoskeletal evaluation or PT treatment	20 (1.5)	14 (2.3)	5 (0.8)	0 (0)	0 (0)	1 (1.3)	
Analgesic treatment	35 (2.6)	26 (6.4)	8 (1.3)	0 (0)	1 (2.9)	0 (0.0)	0.013
**Medications: n (%)**							
ASS 100mg use: any	265 (19.8)	71 (11.8)	168 (28.0)	5 (16.7)	9 (25.7)	12 (16.0)	<0.001
ASS at presentation only	9 (0.7)	3 (0.5)	4 (0.7)	1 (3.3)	0 (0.0)	1 (1.3)	0.38
ASS at discharge only	47 (3.5)	7 (1.2)	40 (6.7)	0 (0.0)	0 (0.0)	0 (0.0)	<0.001
ASS at presentation and discharge	209 (15.6)	61 (10.1)	124 (20.7)	4 (13.3)	9 (25.7)	11 (14.7)	<0.001
Statin use: any	209 (15.6)	62 (10.3)	123 (20.5)	4 (13.3)	11 (31.4)	9 (12.0)	<0.001
Statin at presentation only	7 (0.5)	6 (1.0)	1 (0.2)	0 (0.0)	0 (0.0)	0 (0.0)	0.31
Statin at discharge only	31 (2.3)	11 (1.8)	16 (2.7)	1 (3.3)	0 (0.0)	3 (4.0)	0.58
Statin at presentation and discharge	171 (12.8)	45 (7.5)	106 (17.7)	3 (10.0)	11 (31.4)	6 (8.0)	<0.001
Analgesic use: any	689 (51)	397 (66)	245 (41)	19 (63)	12 (34)	16 (21)	<0.001
Analgesics at presentation only	12 (0.9)	3 (0.5)	6 (1.0)	0 (0.0)	2 (5.7)	1 (1.3)	0.03
Analgesics at discharge only	496 (37.0)	303 (50.3)	166 (27.7)	13 (43.3)	7 (20.0)	7 (9.3)	<0.001
Analgesics at presentation + discharge	181 (13.5)	91 (15.1)	73 (12.2)	6 (20.0)	3 (8.6)	8 (10.7)	0.34
Novel analgesic at discharge for:							
NSAID	312 (23.2)	209 (34.7)	89 (14.9)	11 (36.7)	1 (2.9)	2 (2.7)	<0.001
Paracetamol	407 (30.4)	244 (40.5)	142 (23.7)	9 (30.0)	4 (11.4)	8 (10.7)	<0.001
Opioid	13 (0.9)	8 (1.3)	5 (0.8)	0 (0)	0 (0)	0 (0)	0.85
Metamizole	230 (17.2)	149 (24.8)	67 (11.2)	7 (23.3)	4 (11.4)	3 (4.0)	<0.001
PPI use: any	276 (20.5)	78 (13)	154 (25.7)	5 16.7)	25 (71.4)	14 (18.7)	<0.001
PPI at presentation only	11 (0.8)	4 (0.7)	5 (0.8)	0 (0.0)	2 (5.7)	0 (0.0)	0.02
PPI at discharge only	114 (8.5)	29 (4.8)	65 (10.9)	2 (6.7)	13 (37.1)	5 (6.7)	<0.001
PPI at presentation + discharge	151 (11.3)	45 (7.5)	84 (14.0)	3 (10.0)	10 (28.6)	9 (12.0)	<0.001
Antipsychotic use: any	206 (15.4)	68 (11.3)	108 (18)	6 (20)	5 (14.3)	19 (25.3)	0.002
Antipsychotics at presentation only	17 (1.3)	7 (1.2)	7 (1.2)	1 (3.3)	0 (0.0)	2 (2.7)	0.6
Antipsychotics at discharge only	33 (2.5)	9 (1.5)	17 (2.8)	2 (6.7)	0 (0.0)	5 (6.7)	0.02
Antipsychotics presentation + discharge	156 (11.6)	52 (8.6)	84 (14.0)	3 (10.0)	5 (14.3)	12 (16.0)	0.04
**Recurrent visit: any, n (%)**	176 (13.1)	58 (9.6)	85 (14.2)	11 (36.7)	10 (28.6)	12 (16.0)	<0.001
Outpatient visit	44 (3.3)	11 (1.8)	29 (4.8)	1 (3.3)	2 (5.7)	1 (1.3)	<0.001
Elective hospitalization	21 (1.6)	3 (0.5)	10 (1.7)	4 (13.3)	3 (8.6)	1 (1.3)	
Emergency readmission	108 (8.1)	43 (7.1)	46 (7.7)	4 (13.3)	5 (14.3)	10 (13.3)	
Emergency readmission with hospitalization	3 (0.2)	1 (0.2)	0 (0.0)	2 (6.7)	0 (0.0)	0 (0.0)	
Recurrent visit: related to first ED admission	88 (6.6)	18 (3.0)	48 (8.0)	7 (23.3)	7 (20.0)	8 (10.7)	<0.001
not related / other reasons	88 (6.6)	40 (6.6)	37 (6.2)	4 (13.3)	3 (8.6)	4 (5.3)	
**Outpatient evaluation: n (%)**							
Chest x-ray	40 (3.0)	8 (1.3)	19 (3.2)	9 (30.0)	2 (5.7)	2 (2.7)	<0.001
Chest CT	13 (1.0)	2 (0.3)	4 (0.7)	7 (23.3)	0 (0.0)	0 (0.0)	<0.001
Abdominal CT	4 (0.3)	0 (0.0)	1 (0.2)	2 (6.7)	1 (2.9)	0 (0.0)	<0.001
Abdominal sonography	15 (1.1)	3 (0.5)	7 (1.2)	2 (6.7)	3 (8.6)	0 (0.0)	<0.001
Gastroscopy	10 (0.7)	2 (0.3)	4 (0.7)	0 (0.0)	4 (11.4)	0 (0.0)	<0.001
Coloscopy	2 (0.1)	1 (0.2)	1 (0.2)	0 (0.0)	0 (0.0)	0 (0.0)	<0.001
Treadmill Test	10 (0.7)	0 (0.0)	9 (1.5)	0 (0.0)	1 (2.9)	0 (0.0)	<0.001
Echocardiography	15 (1.1)	3 (0.5)	11 (1.8)	1 (3.3)	0 (0.0)	0 (0.0)	<0.001
MIBI scintigraphy	15 (1.1)	3 (0.5)	12 (2.0)	0 (0.0)	0 (0.0)	0 (0.0)	<0.001
Coronary angiography	8 (0.6)	0 (0.0)	7 (1.2)	0 (0.0)	1 (2.9)	0 (0.0)	<0.001
Pulmonary function test	4 (0.3)	1 (0.2)	2 (0.3)	1 (3.3)	0 (0.0)	0 (0.0)	<0.001
Pleura sonography	4 (0.3)	2 (0.3)	0 (0.0)	2 (6.7)	0 (0.0)	0 (0.0)	<0.001
Surgery	4 (0.3)	1 (0.2)	0 (0.0)	1 (3.3)	2 (5.7)	0 (0.0)	<0.001
Other interventions	36 (2.7)	5 (0.8)	25 (4.2)	3 (10.0)	1 (2.9)	2 (2.7)	<0.001

Values in n (%); p-values refer to all columns except for the overall. Chi-squared tests were used for all variables.MSD, musculoskeletal diseases; GI, gastrointestinal; CVD, cardiovascular disease; PAD, peripheral arterial disease; BMI, body mass index; PPI, proton pump inhibitor; Gyn, gynecological; CT, computer tomography; NSAID, non-steroidal anti-inflammatory drugs; .GP, general practitioner; PT, physical therapist; ASS, acetylic salicylic acid.

**Fig 3:**
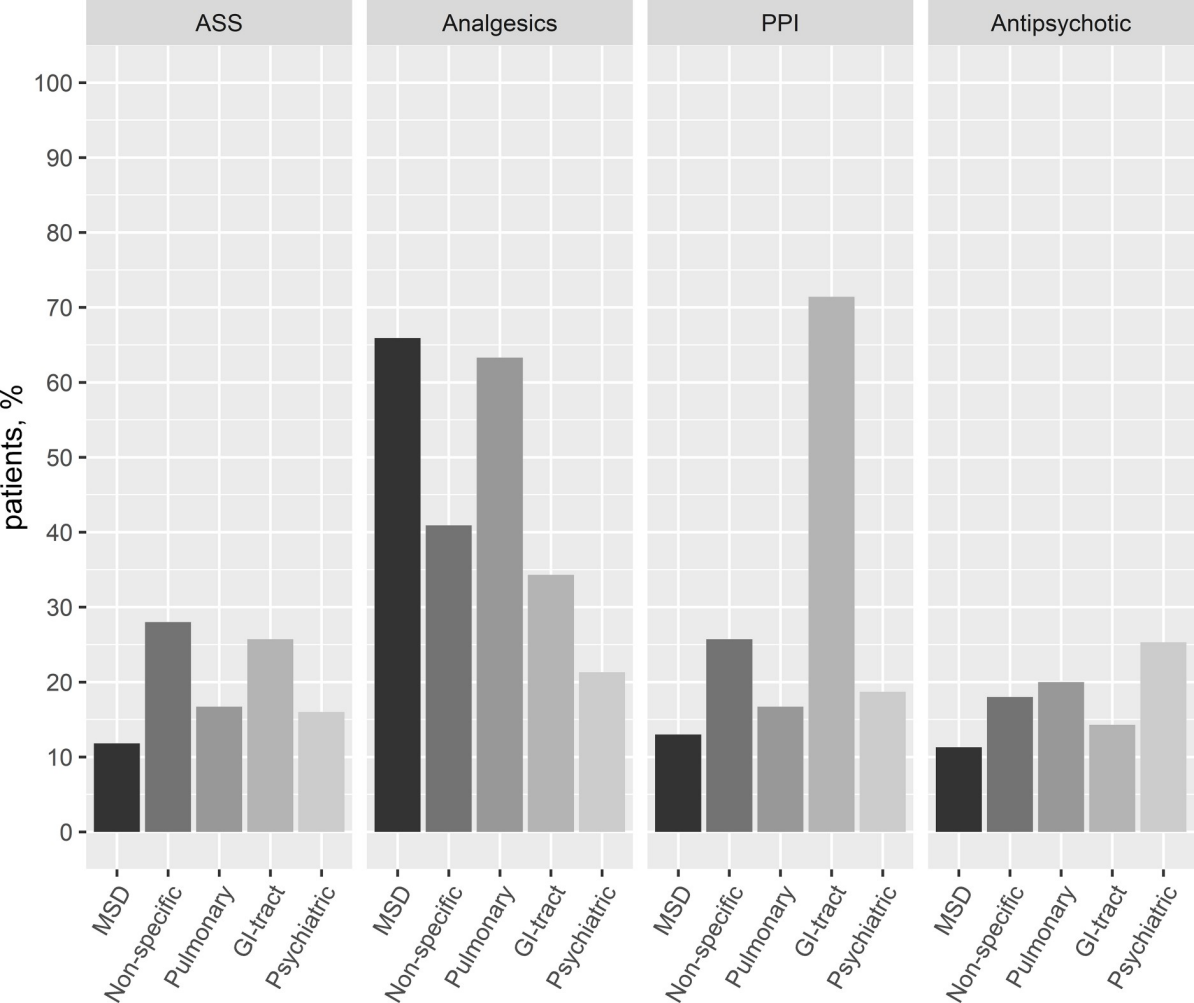
Medications at discharge in non-cardiac chest pain groups. MSD, musculoskeletal chest pain; non-specific, non-specific chest pain; pulmonary, pulmonary diseases; GI-tract, gastrointestinal tract related chest pain; Psychiatric, chest pain related to psychiatric conditions; ASS, acetylsalicylic acid; PPI, proton pump inhibitor.

Newly initiated acetylsalicylic acid (ASS) treatment was found in 3.5% and for statins in 2.3% of patients. Analgesics were used by 51% of all patients (musculoskeletal chest pain category 66%). The most frequently newly prescribed analgesic was paracetamol (30.4%), followed by NSAIDs (23.2%), and metamizole (17.2%). PPI use was found in 20.5% of patients. **Fig 3** shows the differences in the use of medications. The highest proportion of PPI use was found in patients with GI-tract disease (71.4%) and non-specific chest pain diagnoses (25.7%). Antipsychotics were used in 15.4% of the patients with the majority in patients with psychiatric diagnoses (25.3%).

Recurrent visits were recorded in 13.1% mainly with emergency readmissions (8.1%) and outpatient visits (3.3%). Whereas 50% were related to the first emergency visit, 50% were due to other reasons. The highest proportion of related visits was found in patients with pulmonary chest pain (23.3%) and a GI-tract disease (20%). Technical evaluations in the outpatient setting at the same hospital were found in less than 5% of the patients.

## Discussion

The major finding of this study is that chest pain of non-cardiac origin accounted for 4.2% of all ED visits and the diagnostic evaluation included in a minority of patients a formal cardiologic work-up with sequential cardiac troponin testing. In the majority of patients musculoskeletal chest pain or a non-specific chest pain was the discharge diagnosis and psychiatric diseases were rarely considered. Over 72 different recommendations at discharge were given, ranging from no further measures to extensive cardiac evaluation. Despite the recommendation for cardiologic follow-up evaluation in one fifth of the patients, ASS treatment was initiated in only a small proportion of those patients. The most frequently initiated treatment was analgesics where mainly paracetamol was prescribed. A diagnostic test with proton pump inhibitor was prescribed in 20% of patients without specific recommendations about the follow-up assessment.

### Results compared to the literature

NCCP account for a relevant number of emergency department visits. The prevalence of NCCP reported in this study was comparable to a previous study where patients with NCCP accounted for 5% of all ED visits [18]. In the general population, the point prevalence of NCCP may be up to 25% [19]. For example, in a population-based survey in the Olmested County, Minnesota (United States), NCCP was reported by 23% of participants [20]. In patients presenting to the ED with chest pain, 78% consulted a healthcare provider–most commonly general practitioners and cardiologists–in the 12 months previous to the ED presentation [21]. It has been suggested that approximately in two thirds an underlying disease can be identified [22–24].

Proton pump inhibitor treatment trials are highly effective to identify patients with underlying gastroesophageal reflux diseases [15]. Further, panic disorders were common in patients presenting with chest pain to the ED and were rarely recognized by physicians but resulted in more testing and referrals [25]. Therefore, the clinical challenge is to determine which diagnostic tests to apply in patients with chest pain after a cardiac disease has been ruled out to discriminate between patients with non-specific chest pain and other underlying diseases presenting with NCCP. In particular because patients with NCCP experience recurrent pain and a decreased quality of life [26].

This study showed that a primary focus to rule out an acute coronary syndrome (ACS) by extending ECG and cardiac troponin testing to all patients with chest pain in an ED may result in more diagnostic tests without improving the diagnostic and treatment algorithm in the majority of patients. It is important to consider that patients can have elevated troponin levels without cardiac diseases and elevated troponin test may result in more downstream testing without clinical utility [14]. In our study population, no baseline ECG and troponin test was performed in up to 30% and formal ACS rule out testing for fewer than 20%. The diagnoses in those cases was based on the physicians’ clinical assessment of the patients’ history, clinical findings and risk profile. Comparable to our study, in a prospective study of 108 patients with atypical chest pain presenting to an ED in England, treadmill tests (in 9.3% vs. 2.6% in our study), echocardiography (6.5% vs. 4.8%), coronary angiography (4.6% vs. 3%), and gastroscopy (5.6% vs. 1%) were performed in a minority of patients (despite an older mean age of 60 vs. 46 years) [27].

To the best of our knowledge, this was the first study that assessed diagnostic processes and treatment recommendations in patients with NCCP. We found a lack of clinical concepts to assess and treat patients with chest pain after an ACS has been ruled out. Other strategies shown to be effective or useful were not recommended by physicians. For example, a positive response to a high dose PPI treatment trial for one or two weeks indicates an underlying GERD whereas a negative response rules out GERD and can help primary care physicians to further evaluate their patients [15, 28]. According to a Cochrane review cognitive-behavioral therapy may have a short-term effect in patients with chest pain and normal angiogram [29]. However, psychological assessment was recommended in only 3.4% of all patients in this study. Despite the frequency of musculoskeletal chest pain, there is only limited evidence on how to diagnose and treat these patients [30–34]. Physicians use a combination of indicators including the patients’ history and systematic palpation of the spine and chest wall [31]. The treatment strategy found in this study included the prescription of analgesics. Musculoskeletal evaluation and treatment by physical therapists was recommended in 1.5% of the patients. It is unclear what the natural course of patients with musculoskeletal chest pain is and whether more intensive management in some patients may be necessary. In a randomized clinical study that compared manual therapy to self-management, more than one third of the patients complained about chest pain in both groups at the one year follow-up [35].

### Strengths and limitations

While this study was conducted using rigorous predefined protocols and the data extraction quality was high, there are limitations to consider.

The main limitation is the retrospective nature of this study. Patients were identified by ICD-10 codes for non-specific chest pain and therefore, patients with other diseases presenting with chest pain may have been missed. However, the prevalence of patients with NCCP reported in this study was comparable to a previous study [18]. While great care was used when extracting information from the medical charts, we cannot exclude that information was missed during the process despite a data extraction quality (average error rate of below 6%). Our findings have limited generalizability because it is based on data of one teaching hospital and may not apply to other clinical settings. Further, the quality of the discharge diagnosis depends on the clinical experience of a physician and may be revised later on. More experienced physicians may need less diagnostic tests to define a working diagnosis and initiate a treatment. However, residents and an attending physician saw most patients in the hospitals. Therefore, we believe that the study provides an accurate picture of a condition, which in ED department is managed differently compared to primary care practices. The clinical experience of physicians and the skills to communicate with patients may be particularly relevant in patients with non-specific diagnoses including non-cardiac chest pain [13].

### Implications for research

This study has several implications for future research. Future studies should assess the impact of a structured evaluation and treatment recommendation in patients with chest pain after an ACS has been ruled out. To prevent overdiagnosis and overtreatment, studies that assess the efficacy of clinical prediction rules to rule out ACS should be compared to clinical judgment by emergency department physicians. It has been shown that the clinical judgment by ED physicians was at least equally accurate to rule in or out an ACS compared to the HEART score, a prediction rule developed in ED patients with chest pain [36].

### Implications for clinical practice

In patients with non-cardiac chest pain, panic disorders and GERD are rarely considered in the treatment recommendations. This study underscores the need for guidance in patients with non-specific chest pain. Patients with chest pain of unknown or unspecific origin may express avoidance and anxiety symptoms [37]. A structured approach with a defined communication strategy may result in assurance and reduce stress.

### Conclusion

In this retrospective study a formal work-up to rule out ACS was found in a minority of patients presenting to the ED with chest pain of non-cardiac origin. A wide variation in diagnostic processes and treatment recommendations reflect the uncertainty of clinicians on how to approach patients after a cardiac cause is considered unlikely. Panic and anxiety disorders were rarely considered and a useful PPI treatment trial to diagnose gastroesophageal reflux disease was infrequently recommended.

## Supporting information

S1 Table Other preexisting diseases. (DOCX)

S2 Table Troponin testing in patients with non-cardiac chest pain. (DOCX)
