# Supplementary material for: Non-cardiac chest pain patients in the emergency department: Do physicians have a plan how to diagnose and treat them? A retrospective study
Source: PLoS One. 2019 Feb 1;14(2):e0211615. doi: 10.1371/journal.pone.0211615 (PMC6358153; doi:10.1371/journal.pone.0211615)
Supplement: S1 Table — (DOCX) [file pone.0211615.s001.docx]

## Supporting Information 1. Other Preexisting Diseases

|  | **Overall** | **MSD** | **Non-specific** | **Pulmonary** | **GI Tract** | **Psychiatric** | | **p-value** |
| --- | --- | --- | --- | --- | --- | --- | --- | --- |
| Number of patients | 1341 | 602 | 599 | 30 | 35 | 75 | |  |
| Psychiatric diseases | 171 (12.8) | 65 (10.8) | 77 (12.9) | 5 (16.7) | 6 (17.1) | 18 (24.0) | **0.002** | |
| No | 858 (64.0) | 378 (62.8) | 401 (66.9) | 16 (53.3) | 26 (74.3) | 37 (49.3) |  | |
| Not reported | 312 (23.3) | 159 (26.4) | 121 (20.2) | 9 (30.0) | 3 (8.6) | 20 (26.7) |  | |
| GI diseases | 155 (11.5) | 50 (8.3) | 86 (14.4) | 3 (10.0) | 7 (20.0) | 7 (9.3) | **0.01** | |
| No | 885 (66.0) | 401 (66.6) | 395 (65.9) | 19 (63.3) | 24 (68.6) | 46 (61.3) |  | |
| Not reported | 303 (22.6) | 151 (25.1) | 118 (19.7) | 8 (26.7) | 4 (11.4) | 22 (29.3) |  | |
| Known cancer disease | 31 (2.3) | 14 (2.3) | 13 (2.2) | 2 (6.7) | 2 (5.7) | 0 (0.0) | 0.05 | |
| No | 1000 (74.6) | 432 (71.8) | 467 (78.0) | 20 (66.7) | 28 (80.0) | 53 (70.7) |  | |
| Not reported | 310 (23.1) | 156 (25.9) | 119 (19.9) | 8 (26.7) | 5 (14.3) | 22 (29.3) |  | |
| Thyroids disease | 55 (4.1) | 21 (3.5) | 27 (4.5) | 1 (3.3) | 1 (2.9) | 5 (6.7) | 0.2 | |
| No | 979 (73.0) | 427 (70.9) | 451 (75.3) | 22 (73.3) | 30 (85.7) | 49 (65.3) |  | |
| Not reported | 307 (22.9) | 154 (25.6) | 121 (20.2) | 7 (23.3) | 4 (11.4) | 21 (28.0) |  | |
| Pulmonary disease | 95 (7.1) | 45 (7.5) | 42 (7.0) | 3 (10.0) | 1 (2.9) | 4 (5.3) | 0.08 | |
| No | 941 (70.2) | 401 (66.6) | 441 (73.6) | 19 (63.3) | 30 (85.7) | 50 (66.7) |  | |
| Not reported | 305 (22.7) | 156 (25.9) | 116 (19.4) | 8 (26.7) | 4 (11.4) | 21 (28.0) |  | |
| Gyn./urological disease | 84 (6.3) | 24 (4.0) | 51 (8.5) | 3 (10.0) | 2 (5.7) | 4 (5.3) | **0.008** | |
| No | 948 (70.7) | 421 (69.9) | 430 (71.8) | 19 (63.3) | 29 (82.9) | 49 (65.3) |  | |
| Not reported | 309 (23.0) | 157 (26.1) | 118 (19.7) | 8 (26.7) | 4 (11.4) | 22 (29.3) |  | |
| Rheumatic disease | 33 (2.5) | 10 (1.7) | 17 (2.8) | 2 (6.7) | 3 (8.6) | 1 (1.3) | **0.006** | |
| No | 995 (74.2) | 434 (72.1) | 461 (77.0) | 20 (66.7) | 29 (82.9) | 51 (68.0) |  | |
| Not reported | 313 (23.3) | 158 (26.2) | 121 (20.2) | 8 (26.7) | 3 (8.6) | 23 (30.7) |  | |
| Other | 382 (28.5) | 157 (26.1) | 189 (31.6) | 10 (33.3) | 11 (31.4) | 15 (20.0) | 0.14 | |
| No | 676 (50.4) | 303 (50.3) | 299 (49.9) | 14 (46.7) | 20 (57.1) | 40 (53.3) |  | |
| Not reported | 283 (21.1) | 142 (23.6) | 111 (18.5) | 6 (20.0) | 4 (11.4) | 20 (26.7) |  | |
| Multi morbidity* | 429 (32.0) | 139 (23.1) | 237 (39.6) | 13 (43.3) | 17 (48.6) | 23 (30.7) | **<0.001** | |

Values in median [IQR], n (%); p-values refer to all columns except for the overall. A chi-squared test was used for all variables except Age and BMI where Kruskal-Wallis was used.

MSD, musculoskeletal diseases; GI, gastrointestinal; CVD, cardiovascular disease; PAD, peripheral arterial disease; BMI, body mass index; PPI, proton pump inhibitor; Gyn, gynecological; not reported, no information available in the electronical records.

*≥2 morbidities: PAD, cardiovascular diseases or previous acute MI, diabetes mellitus, antihypertensive therapy, psychiatric diseases, gastrointestinal diseases, known cancer diseases, thyroids disease, pulmonary disease, gynecological/urological disease, rheumatic disease, other diseases
