# Supplementary material for: Non-cardiac chest pain patients in the emergency department: Do physicians have a plan how to diagnose and treat them? A retrospective study
Source: PLoS One. 2019 Feb 1;14(2):e0211615. doi: 10.1371/journal.pone.0211615 (PMC6358153; doi:10.1371/journal.pone.0211615)
Supplement: S2 Table — (DOCX) [file pone.0211615.s002.docx]

## Supporting Information 2. Troponin testing in patients with non-cardiac chest pain

|  | **Overall** | **MSD** | **Non-specific** | **Pulmonary** | **GI-tract** | **Psychiatric** | **p** |
| --- | --- | --- | --- | --- | --- | --- | --- |
| Patients: n | 1341 | 602 | 599 | 30 | 35 | 75 |  |
| Troponin test at presentation | 1091 (81.4) | 439 (72.9) | 547 (91.3) | 18 (60.0) | 32 (91.4) | 55 (73.3) | **<0.001** |
| Not measured | 250 (18.6) | 163 (27.1) | 52 (8.7) | 12 (40.0) | 3 (8.6) | 20 (26.7) |  |
| Troponin 2° within 6 hours | 558 (11.6) | 168 (27.9) | 349 (58.3) | 4 (13.3) | 23 (65.7) | 14 (18.7) | **<0.001** |
| Troponin 2° ≥6 hours | 14 (1.3) | 5 (0.8) | 6 (1.0) | 2 (6.7) | 0 (0.0) | 1 (1.3) |  |
| Troponin 3° between 6 and 12 hours after presentation, n (%) | 54 (1.0) | 11 (2.5) | 39 (6.5) | 1 (3.3) | 3 (8.6) | 0 (0.0) | **0.001** |
| Troponin 3° ≥12 hours after presentation, n (%) | 36 (2.7) | 8 (1.3) | 25 (4.2) | 1 (3.3) | 1 (2.9) | 1 (1.3) | **0.042** |
| Troponin 3° <6 hours after presentation, n (%) | 33 (2.5) | 9 (1.5) | 20 (3.3) | 1 (3.3) | 1 (2.9) | 2 (2.7) | **0.355** |

Time between test were calculated by subtracting the time stamps of the troponin test 1 from the test 2 and 3.
